# Peripheral Neuropathy and VIth Nerve Palsy Related to Randall Disease Successfully Treated by High-Dose Melphalan, Autologous Blood Stem Cell Transplantation, and VIth Nerve Decompression Surgery

**DOI:** 10.1155/2010/542925

**Published:** 2010-12-16

**Authors:** C. Foguem, P. Manckoundia, P. Pfitzenmeyer, J.-L. Dupond

**Affiliations:** ^1^Service de Gériatrie, Hôpital Jean Minjoz, CHU, Boulevard Fleming, 25000 Besançon Cedex, France; ^2^Service de Médecine Interne Gériatrie, Hôpital de Champmaillot, CHU BP 87909, 2 rue Jules Violle, 21079 Dijon Cedex, France; ^3^INSERM/U887 Motricité-Plasticité: Performance, Dysfonctionnement, Vieillissement et Technologies d'optimisation, Université de Bourgogne, BP 27877, 21078 Dijon Cedex, France; ^4^Service de Médecine Interne, Hôpital Jean Minjoz, CHU, Boulevard Fleming, 25000 Besançon Cedex, France

## Abstract

Randall disease is an unusual cause of extraocular motor nerve (VI) palsy. A 35-year-old woman was hospitalized for sicca syndrome. The physical examination showed general weakness, weight loss, diplopia related to a left VIth nerve palsy, hypertrophy of the submandibular salivary glands, and peripheral neuropathy. The biological screening revealed renal insufficiency, serum monoclonal kappa light chain immunoglobulin, urinary monoclonal kappa light chain immunoglobulin, albuminuria, and Bence-Jones proteinuria. Bone marrow biopsy revealed medullar plasma cell infiltration. Immunofixation associated with electron microscopy analysis of the salivary glands showed deposits of kappa light chains. Randall disease was diagnosed. The patient received high-dose melphalan followed by autostem cell transplantation which led to rapid remission. Indeed, at the 2-month followup assessment, the submandibular salivary gland hypertrophy and renal insufficiency had disappeared, and the peripheral neuropathy, proteinuria, and serum monoclonal light chain had decreased significantly. The persistent diplopia was treated with nerve decompression surgery of the left extraocular motor nerve. Cranial nerve complications of Randall disease deserve to be recognized.

## 1. Introduction

Randall disease (RD) is characterized by tissue deposition of monoclonal immunoglobulin light chains without tinctorial properties [1]. We report a case of RD associated with plasma cell dyscrasia, left VIth nerve palsy, peripheral neuropathy, kidney disease, and submandibular salivary gland hypertrophy.

## 2. Case Report

**Figure 1:**
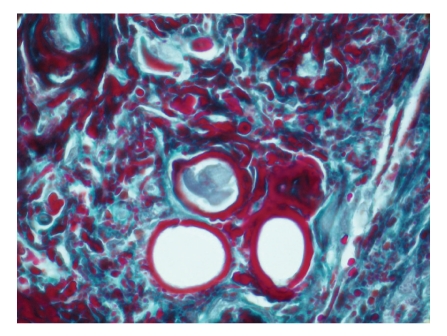
Immunohistologic analysis of submandibular salivary gland biopsy showing deposits of light chain monoclonal immunoglobulin in the perivascular space and connective tissues. Deposits are brick-red after Masson's Trichrome stain.

A 35-year-old woman was hospitalized for sicca syndrome lasting for 6 months. In addition to general weakness and a 6 kg weight loss, the physical examination showed diplopia related to left VIth nerve palsy as confirmed by the ophthalmological examination, submandibular salivary gland enlargement, and peripheral neuropathy confirmed by the electromyogram. Biological screening revealed moderate renal insufficiency with creatinine clearance at 47 mL/min/1.73 m^2^, serum monoclonal kappa light chain immunoglobulin with a level of 175 mg/L and a kappa/lambda ratio of 49, urinary monoclonal kappa light chain immunoglobulin, and proteinuria at 2 g/24 hours with positive Bence-Jones proteinuria. Bone marrow biopsy revealed medullar plasma cell infiltration representing up to 20% of medullar cells. However, there were no other criteria for multiple myeloma. Immunofixation associated with electron microscopy analysis of the salivary glands showed deposits of kappa light chains without characteristics of amyloidosic proteins (Figure 1). In light of these abnormalities, RD associated with plasma cell dyscrasia, left VIth nerve palsy, peripheral neuropathy, kidney disease, and submandibular salivary gland hypertrophy was diagnosed. The patient received high dose melphalan (HDM) (200 mg/m^2^) followed by autostem cell transplantation (SCT) (CD 34 × 10^6^/kg) which resulted in rapid subtotal and persistent remission. Indeed, two months after the treatment, the submandibular salivary gland hypertrophy had disappeared, the general state of health and peripheral neuropathy had improved, renal function had returned to normal with an increase in creatinine clearance to 91 mL/min/1.73 m^2^ and a decrease in proteinuria (<1 g/24 hours), the serum monoclonal light chain level stood at 9.66 mg/L, and the kappa/lambda ratio was 1.97. However, there was still dysaesthesia of the left hand and left VIth nerve palsy. The latter was treated with nerve decompression surgery with disappearance of diplopia one year later. At the 3-year followup assessment, there was no recurrence, but only a persistence of slight paresthesia of the left hand.

## 3. Discussion

Randall disease is a monoclonal immunoglobulin deposition disease [2]. Monoclonal immunoglobulin deposition disease is a systemic disorder with immunoglobulin chain deposition in a variety of organs, leading to various clinical features [3]. Visceral immunoglobulin chain deposits may be totally asymptomatic and found only at autopsy [4]. Submandibular salivary glands can be affected by monoclonal immunoglobulin deposition disease (MIDD). However, peripheral neuropathy and cranial nerve palsies in general, and extraocular motor nerve (VI) palsy associated with diplopia in particular, in the context of RD, are rarely reported in the literature. In 1998, Grassi et al. reported the first precise morphologic and clinical description of neuropathy related to RD [5]. 

The diagnosis of monoclonal immunoglobulin deposition disease must be suspected in front of nephrotic syndrome, rapidly progressive tubulointerstitial nephritis, or echocardiographic findings indicating diastolic dysfunction and the discovery of a monoclonal immunoglobulin component in the serum and/or the urine [4]. The definitive diagnosis is obtained by the immunohistologic analysis of the biopsy of an affected organ, mainly the kidney, using a panel of immunoglobulin chain-specific antibodies, including anti-*κ* and anti-*λ* light chain antibodies to stain the non-Congophilic deposits [4]. In our paper, the diagnosis was made by the immunohistologic analysis of the salivary glands. There is no standard treatment for RD [6, 7]. Recent publications have emphasized the success of HDM/auto-SCT [6] which now appears to be the most reliable and effective treatment of neurological complications of MIDD in young patients. Indeed, the literature reports the successful treatment of AL amyloid polyneuropathy with this therapy [8]. Novel therapies—thalidomide, bortezomib, and lenalidomide—used in myeloma have not been sufficiently studied in RD [9]. The future prospects for therapy are based on the pathophysiology of RD and include the blocking of light chain binding to mesangial receptors, the use of transforming growth factor beta (TGF-*β*) antagonists and inhibitors of light chain-induced signalling pathways [4].

This paper is educational in that it demonstrates the interest of considering RD in a clinical picture of a cranial nerve disorder. Further analyses will confirm the diagnosis, and appropriate therapy can improve the clinical abnormalities and prevent potentially serious functional complications. Finally, because of the rarity of this pathology and the improvement in symptoms obtained with high-dose melphalan with auto-SCT and nerve decompression surgery in this young patient, this paper could contribute to the medical literature which is currently scarce in this disease.
